# *In Silico*, Computer Simulations from Neurons up to the Whole Brain

**DOI:** 10.1523/ENEURO.0124-21.2021

**Published:** 2021-04-27

**Authors:** Alain Destexhe

**Affiliations:** Paris-Saclay University, Centre National de la Recherche Scientifique, 91198 Gif sur Yvette, France

**Keywords:** computational models, neurons, large-scale models, numerical simulations, neural networks

## Abstract

This commentary puts the *In Silico* movie in perspective of the Human Brain Project (HBP) and clarifies major differences between this project and the Blue Brain Project, emphasizing that the two projects are very different in scope.

The *In Silico* movie has retraced, quite accurately, the time line of the construction of detailed brain models, incarnated by the Blue Brain Project headed by Henry Markram. The movie starts from the beginning of the project and goes up to 2020, >10 years after its beginning. The movie also talks about the “excursion” of a couple years into the Human Brain Project (HBP).

The movie is of course very centered on Henry Markram, who is the initiator of this line of research. It presents quite well how he has initiated and conceived the Blue Brain adventure. Almost naturally, the HBP is also presented as a pure creation of Henry, from its beginning. As one of the founding members of the HBP, I would like to provide some corrections and precisions about some misleading statements in the movie, and in particular about his interaction with the HBP, and the very different goals of the two projects.

As rightly said by Idan Segev, Henry is a visionary scientist, and he has been the initiator of the HBP in the sense that the initial idea, the initial vision, came from him. However, the movie presents the “EPFL view” of the creation of the HBP and masks a lot of work and other European researchers who had a decisive role in building the HBP, and I would like to clarify some of the points that are perhaps too quickly or too simply described in the movie.

A first point is that there has been a big confusion by various people and media, who wrongly stated that the goal of the HBP is to simulate the entire human brain at cellular resolution. This is clearly a stated goal of the Blue Brain Project and may also be the goal of some subproject within the HBP (such as the one led by Henry), but it is wrong to say that this is the goal of the HBP. If you read the HBP project, and its plan for 10 years available online (The Human Brain Project, A report to the European Commission, 2012), you will see that, indeed, a detailed simulation of the human brain is proposed. But this represents only a small part of the HBP, perhaps 10% in terms of budget and human effort. My personal impression is that, because this goal has a very provocative aspect, it was taken up by those who only superficially (or not at all) read the project, or by some news media. But this ignores the remaining 90% of the project!

The goal of the HBP, as initially stated, is in short to “better understand the brain and its pathologies using information technologies” (1). This includes far more than the Blue Brain-like approach of simulating extremely complex networks of neurons. It includes studying simplified models of neurons, neural networks, population models, deep learning approaches, etc. There is also a large part (actually the largest in terms of budget) devoted to technological developments, like neuromorphic engineering, simulation engines, databases, brain atlases, neurorobotics, medical informatics, etc. It is so frustrating for researchers participating to the HBP to see that these important areas of research are often passed under silence by the media, especially from those who criticize the project. It is like saying that one should not have funded this project because 10% of the project is subject to controversy.

This imbalance is also seen in the movie, which focuses on Henry Markram’s role in the HBP during nearly 20 min, and only mentions the rest of the HBP in a couple seconds!

The movie also retraces very well the criticisms of the community against the Blue Brain Project and the HBP. However, the criticisms against HBP have essentially focused on Henry Markram and very little on the rest of the HBP (an important point not mentioned at all in the movie, unfortunately). The movie could have made the point that those who criticize the HBP, focusing on Markram, and ignoring 90% of the project, are obviously not motivated by science, because if it was so, the critics would have read the HBP and realized that the Blue Brain-like approach is only a small part of it. In my opinion, such criticisms are rather driven by ego considerations, or personal conflicts with Henry Markram. The Blue Brain Project was also highly criticized by the scientific community in Switzerland, a fact that the movie could have better described as well.

Second, the movie describes a leading Henry’s role in constructing the HBP. Indeed, Henry had the initial vision, but the construction of HBP was the fruit of merging different projects across Europe. Besides the “BlueBrain stream” (Henry Markram and various associated labs collaborating with him), very well described in the movie, there was also the “BrainScales stream,” to which I belong. Unfortunately, this was not described in the movie. This stream was a group of European labs (Karlheinz Meier, Yves Frégnac, Wolfgang Maass, Steve Furber, Ad Aertsen, and Wulfram Gerstner, among others), who obtained a series of successive European projects [SenseMaker (The SenseMaker Project, 2002), FACETS (The FACETS Project, 2005; EU FET Open Integrated project, BioI3 program, FP6-015879), BrainScales (The BrainScales project, 2011), etc.]. The main line in these projects was to associate experimental and theoretical neuroscience with technological developments such as neuromorphic computers (see also description in Frégnac and Laurent, 2014). The general idea was to extract principles from experimental data, formalize these principles into equations and models, and implement these models in dedicated circuits to build machines that perform bio-inspired brain computations. Nearly all the partners of these projects ended up in the HBP, and this stream of research can be found, almost intact, in the HBP since its beginning and is still there today. Karlheinz Meier was the co-director of HBP for several years, before he unfortunately passed away in 2018, and he was a coordinator of these integrated projects. Curiously, this stream of research is not described in the movie, although its overall budget represents the major part of the HBP, it had few critics if any, and it is still present today.

Third, the movie described a little bit too briefly the dispute with the cognitive neuroscience in the HBP, and in particular involving Stanislas Dehaene. For reasons that are still obscure today, the “triumvirat” (Markram, Meier, Frackowiak) decided to expel the cognitive neuroscience part of the project (coordinated by Dehaene). This led to an internal fight, where most of the neuroscientists in the HBP supported cognitive neuroscience, but the decision was forced by the three co-directors. This happened at a meeting that I hosted at the European Institute of Theoretical Neuroscience in Paris in March 2014 (European Institute of Theoretical Neuroscience, 2014), where the vote occurred. This decision of expelling cognitive neuroscience, against nearly the half of the project, is what caused a great damage in terms of relation between HBP and the neuroscience community.

This expulsion, and the open letter that followed, was described in the movie and triggered a reaction by the European Community unit driving the project. It was realized that a triumvirat is inappropriate for such a large project, and following a mediation process, a new governance was installed, more collegial and bottom-up driven, comprising initially 12 panel members (I represented the theoretical neuroscience among these 12 members). It was also decided to reintegrate cognitive neuroscience, through an “open call” for partners, who could join the project after being selected by a scientific review panel organized by the EU. Finally, the board of directors elected a chairperson, Katrin Amunts, who is still acting chair of the Scientific and Advisory Board of the project.

This marked the end of the “Markram era” in the HBP, and a *de facto* separation between the HBP and the Blue Brain Project, which was also asked for by the EU.

Unfortunately, this situation is not well described in the movie, as well as in other news media, which tend to focus on the neuroscience community opposing Henry’s claim to simulate the human brain, while the reality is more complicated.

It is important to note that, although Henry Markram is not anymore today a leader in the HBP, the project owes him a lot. It seems clear, at least to me, that the HBP could never have been created without Henry. He was not only the initiator of the project, but he also masterfully defended the project during its audition and review by the EU. One may agree or disagree with his science, but he is a true visionary person and able to gather and convince people to join him. These qualities were essential to make the HBP selected as a European Flagship.

Fourth, the movie retraces very well some of the critics of the “bottom-up” modeling strategy followed in the HBP and Blue Brain Project, but this criticism is delivered without any context or perspective. In reality, the dilemma between bottom-up or top-down strategies is very old in neuroscience, and while Zach Mainen’s criticism and image of the watch is well taken, it is inaccurate. Bottom-up strategies constitute the basis of statistical physics types of approaches, which attempt to explain the macroscopic properties of matter (such as solid, liquid or gas states, turbulence, etc.) from the properties of molecules in interaction. In computational neuroscience, these statistical physics types of approaches are also followed and try to understand the emergence of macroscopic brain states (wake, sleep, anesthesia, pathologies) from neurons and their interaction. Of course, it is right to say that to reconstruct a watch one needs all of its constituents, which is not the case in neurons where many constituents are still unknown. However, it is wrong to say that the bottom-up approach is flawed for this reason. There are many successful stories in computational neuroscience where emerging properties of networks (such as decision-making, different brain states, illusions, etc.) could be understood as emerging from network interactions between simplified neurons. The same approach is also followed in the HBP, where the bottom-up strategy is based on simplified neuron models (such as the “integrate and fire” neuron).

Fifth, the movie accounts well in detail of the Blue Brain-type simulations in the HBP, but it does not mention that another type of brain simulation, using more macroscopic or population type models, is investigated in the HBP. Rather than being based on cellular-level models, these whole-brain simulations are based on population (mean-field) models each representing a tiny brain region. The main reason is that such whole-brain models are typically constrained by imaging data, or mesoscopic measurements such as local field potentials, voltage-sensitive dye imaging or calcium imaging, and these signals are all population signals, so there would be no point of simulating models at cellular scale to model such data. Indeed, the whole-brain models (Deco and Kringelbach, 2014) can investigate the genesis of large-scale phenomena such as slow waves and how the brain responds to external stimuli (Goldman et al., 2020). In some cases, they can run on a laptop computer, so this type of whole-brain models does not require aberrant computational resources.

But more importantly, building whole-brain models has never been a goal in itself for the HBP but is rather a tool to study the dynamics of the human brain at large scales, based on global signals such as neuroimaging, electroencephalogram or magnetoencephalogram signals. So, simulating the human brain is still planned in the HBP but doing so at cellular resolution was abandoned, while this cellular-resolution effort continues in the Blue Brain Project. This main difference, which reflects today’s situation, was unfortunately not well explained in the movie.

In conclusion, the movie *In Silico* is certainly one of the most detailed having been done on the Blue Brain Project. However, its description of the interaction between the Blue Brain and the HBP is too simplistic [see also associated commentaries by Viktor Jirsa (Jirsa, 2021) and Yves Frégnac (Frégnac, 2021)]. I hope that this commentary will clarify a little bit the relations between these two fascinating projects.

## Footnotes

^1^The exact formulation was “The Human Brain Project should lay the technical foundations for a new model of ICT-based brain research, driving integration between data and knowledge from different disciplines, and catalysing a community effort to achieve a new understanding of the brain, new treatments of brain disease and new brain-like computing technologies.”

## Synthesis

Reviewing Editor: Christophe Bernard, INSERM & Institut de Neurosciences des Systèmes Decisions are customarily a result of the Reviewing Editor and the peer reviewers coming together and discussing their recommendations until a consensus is reached. When revisions are invited, a fact-based synthesis statement explaining their decision and outlining what is needed to prepare a revision will be listed below. The following reviewer(s) agreed to reveal their identity: NONE.

na
